# Detection of *Anaplasma phagocytophilum* in *Ixodes ricinus* ticks from Norway using a realtime PCR assay targeting the Anaplasma citrate synthase gene *gltA*

**DOI:** 10.1186/s12866-015-0486-5

**Published:** 2015-08-01

**Authors:** Anna J. Henningsson, Dag Hvidsten, Bjørn-Erik Kristiansen, Andreas Matussek, Snorre Stuen, Andrew Jenkins

**Affiliations:** Department of Clinical Microbiology, Division of Medical Services, County Hospital Ryhov, Jönköping, Sweden; Department of Microbiology and Infection Control, University Hospital of North Norway, Tromsø, Norway; Research group of host-microbe interactions, Department of Medical Biology, Faculty of Health Sciences, UiT - The Arctic University of Norway, Tromsø, Norway; Department of Production Animal Clinical Sciences, Norwegian University of Life Sciences, Sandnes, Norway; Department of Environmental and Health Studies, Telemark University College, Bø, Norway

**Keywords:** *Anaplasma phagocytophilum*, *Ixodes ricinus*, TaqMan realtime PCR, Norway, *gltA*, Prevalence

## Abstract

**Background:**

A TaqMan real-time PCR assay targeting the *Anaplasma* citrate synthase gene, *gltA*, was developed and used for detection of *Anaplasma phagocytophilum* in 765 *Ixodes ricinus* ticks collected from dogs and cats in northern Norway (*n* = 669) and Telemark county in southern Norway (*n* = 96).

**Results:**

Among the ticks from northern Norway the prevalence of *A. phagocytophilum* was 3.0 %, while the prevalence in southern Norway was 2.1 % (*p* = 0.63). The *gltA* PCR assay showed a high analytical sensitivity (30 genomic units) and efficiency (98.5 %), and its utility in clinical diagnostics should be evaluated in future studies.

**Conclusion:**

This is the first report of *A. phagocytophilum* occurrence in ticks collected north of the Arctic Circle in Norway. The prevalence is comparable to that found in Telemark county in southern Norway.

## Background

*Anaplasma phagocytophilum* is an obligate intracellular rickettsial pathogen transmitted by *Ixodes* ticks [1]. The bacterium is a well-documented pathogen in veterinary medicine, causing tick-borne fever (TBF) in domestic ruminants and horses [2, 3]. The first verified cases of human granulocytic anaplasmosis (HGA) were reported from the USA in 1994 [4, 5], and the first verified European cases were reported from Slovenia in 1997 [6]. HGA cases have been reported from southern Norway and there is serological evidence of *Anaplasma* endemicity [7–10]. The pathogen is also prevalent in Norwegian livestock [11], and serological findings in cattle have indicated the presence of *A. phagocytophilum* in Nordland county in northern Norway [12]. *Anaplasma phagocytophilum* is a possible emerging tick-borne pathogen in northern Norway, but reports on human disease are lacking.

The prevalence of *A. phagocytophilum* infection in the European tick *Ixodes ricinus* varies in different areas and between developmental stages of the tick [13]. In Norway, recent studies indicate a prevalence in host-seeking *I. ricinus* of 1.4 % to 19.4 % with great regional differences [9, 14, 15].

*In vitro* cultivation and blood smear microscopy are difficult and cumbersome methods for detection of *A. phagocytophilum* in clinical samples, and therefore clinical diagnostics is mainly based on serology and PCR methods [16]. Major advantages of PCR methods are rapid test results, high sensitivity and the possibility of quantifying pathogen load. Several PCR assays have been described for the detection of *A. phagocytophilum* in clinical samples and in ticks [17–19]. Real time PCR offers gains of rapidity and quantitativity and runs in a closed-system format, which eliminates risks of carry-over contamination. The *A. phagocytophilum* gene *gltA* codes for an essential housekeeping enzyme, citrate synthase. Mutations in such genes are predominantly point mutations that conserve enzyme function and they are not subject to the effects of diversifying selection that may affect surface proteins targeted by the immune system. This makes them attractive targets for phylogenetic studies, making extensive high-quality sequence information available. They are less conserved than 16 s rRNA genes and generally lack secondary structure, which simplifies the design of species-specific PCR.

In this study, we developed a TaqMan real-time PCR assay targeting *gltA* for the detection of *A. phagocytophilum*. Using this test, we aimed at extending previous studies at the ticks’ northern distribution limit by investigating the prevalence of *A. phagocytophilum* in *I. ricinus* ticks collected during a three-year period from different locations in northern and southern Norway.

## Methods

### Study area and design

**Fig. 1:**
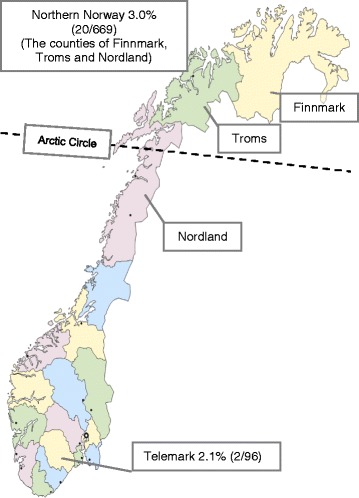
The study areas. The study areas in northern Norway (the counties of Nordland, Troms and Finnmark) and in southeastern Norway (the county of Telemark). Prevalence of *A. phagocytophilum* in the collected ticks is shown

Collection of ticks from dogs and cats was previously described [20, 21]. Briefly, during 2009, 28 veterinary clinics in the three northernmost Norwegian counties of Nordland, Troms and Finnmark (*n* = 23), and in the southern county of Telemark (*n* = 5) collected ticks from dogs and cats [20]. In 2010–2011, veterinary clinics in districts in northern Norway (Nordland, *n* = 7; Troms, *n* = 1) collected ticks from dogs and cats for one season each (Fig. 1) [21]. For each tick delivery, data concerning number of ticks, the geographical origin and kind of source (dog, cat or other sources) was collected. Ticks were placed in plastic tubes containing 1 mL to 3 mL 70 % ethanol and kept at 4° to 8 °C until analysis. The collected ticks were examined by stereo light microscopy for determination of species and instar. Only *I. ricinus* ticks were included. Ticks from dogs or cats that had been outside the study areas during the preceding ten days were excluded. One tick only was collected by the veterinary clinics in Finnmark, but had to be excluded due to the pet’s recent travel outside the study area. Ticks from other sources than dogs or cats were not included. To avoid prevalence overestimates due to co-feeding [22], the pet source and PCR findings were scrutinised, and if there was a possibility of co-feeding and PCR results were concordant, the ticks were not included.

### Nucleic acid extraction and design of *A. phagocytophilum* PCR

Nucleic acid extraction was done as previously described [20], individually from each tick and stored at – 20 °C until use.

Multiple sequence alignment [23] of six *gltA* sequences of *A. phagocytophilum* with a selection of the most similar *gltA* sequences from related species (*A. platys*, *Rickettsia africae*, *R. heilongjiangensis*, *R. principis* and *Wolbachia* DQ266529) identified by BLAST search (http://blast.ncbi.nlm.nih.gov/) revealed a sequence region from 320–435 in the reference sequence AF304137 that was both highly conserved within *A. phagocytophilum* and highly divergent in related species. Primer-Express (Applied Biosystems, Foster City, CA) was used to design a two-step TaqMan MGB® PCR targeting a 64 bp segment of this region. Table 1 lists the primers, probe and cycling parameters. Every reaction consisted of 25 μL containing 12.5 μL 2x Taqman Universal PCR mastermix (Life Technologies, Waltham, MA, USA), 600 nM of each primer, 150 nM probe, 4.5 μL RNAse free water and 5 μL DNA. Primers and probe were obtained from Life Technologies. The probe incorporated a stabilising minor groove binder (MGB®) and was labelled with fluorescein amidite (FAM) and equipped with a dark quencher.

**Table 1:** Primers, probes and cycling parameters

Name	Sequence	Function	Tm	Concentration
ApF	TTTTGGGCGCTGAATACGAT	Forward Primer	59 °C	300 nM
ApR	TCTCGAGGGAATGATCTAATAACGT	Reverse Primer	58 °C	300 nM
ApM	TGCCTGAAC AAGTTATG	5’ hydrolysis probe	69 °C	300 nM

Cycling parameters: 50 °C, 10 min; 95 °C, 2 min; {95 °C, 15 s; 60 °C, 60s} × 40 cycles

The initial 50 °C incubation is an optional step allowing the decontaminating action of uracyl nucleoside glycosylase (UNG), if present

A synthetic plasmid, pAP-GltA, containing the amplicon sequence cloned in pUC57 was obtained from Genscript Corporation (Scotch Plains, NJ). This plasmid was used as a positive control. Serial dilutions of the plasmid were used to determine analytical sensitivity and as a quantitation standard.

### Statistics

Differences in *A. phagocytophilum* prevalence were analysed by Chi-Square test, *p* < 0.05 was regarded as significant.

### Ethics

No ethical approval was required for this study since removal of ticks was part of routine care of the pets.

## Results

### Collected ticks

In total, 765 *I. ricinus* ticks were collected; 669 from northern Norway (Nordland *n* = 647, Troms *n* = 22, Finnmark *n* = 0) and 96 from Telemark county in southern Norway. Four hundred-and-sixty-three (61 %) were detached from dogs (*n* = 330), and 302 (39 %) from cats (*n* = 190) (Table 2). Of the collected ticks, 690 were adult females, of which approximately 90 % were moderately to fully engorged; there were 69 adult males, four nymphs and two ticks of undetermined instar.

**Table 2:** Collected ticks, their origin and prevalence of *Anaplasma phagocytophilum*

	No. of *A. phagocytophilum-*infected ticks (%)	No. of *A. phagocytophilum-*infected ticks from dogs (%)	No. of *A.* *phagocytophilum*-infected ticks from cats (%)
Troms	2/22 (9.1)	2/10 (20)	0/12 (0)
Nordland	18/647 (2.8)	11/361 (3.0)	7/286 (2.4)
**Total North Norway**	**20/669 (3.0)**	**13/371 (3.5)**	**7/298 (2.3)**
Telemark	2/96 (2.1)	2/92 (2.2)	0/4 (0)
**Total**	**22/765 (2.9)**	**15/463 (3.2)**	**7/302 (2.3)**

### In silico evaluation of the PCR test

**Fig. 2:**
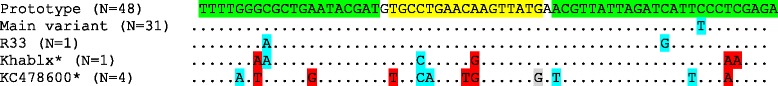
The PCR target region. Multiple sequence alignment of variants of the PCR target sequence. The positions of the primers and probe targets are highlighted in green and yellow respectively. Dots indicate identity to the prototype sequence. Variant nucleotides are shown as highlighted letters. Blue highlighting indicates variations that make non-destabilizing G:T base pairing with the primer/probe. Red highlighting indicates destabilizing mismatches. Variant nucleotides outside the primer/probe targets are highlighted in grey. *: isolates of exclusively far-eastern origin

A BLAST search conducted in June 2015 (http://blast.ncbi.nlm.nih.gov/) using the entire 64 bp amplicon sequence gave 85 hits to *A. phagocytophilum gltA* sequences. Fourty-eight sequences showed 100 % similarity to the primer and probe sequences, 31 showed a single mismatch in the reverse primer and one (R33, JX841253) showed single mismatches in central parts of both primer sequences. Both these mismatches will result in stable, non-canonical G:T basepairs and are unlikely to appreciably affect PCR sensitivity. Five sequences, all of far-eastern origin, were highly divergent and are unlikely to be detected efficiently, if at all. A comparison of the sequences is shown in Fig. 2. Hits to non-*A. phagocytophilum* sequences were short and/or highly mismatched. A high degree of specificity for *A. phagocytophilum* is thus expected.

### Analytical sensitivity and efficiency of the gltA PCR assay

Analysis of a 10x serial dilution of pAP-GltA from 3.10^6^ to 30 genomic units (GU)/5 μL (in duplicate) using the analysis software of the Applied Biosystems StepOne genetic analyzer gave a PCR efficiency of 98.5 % based on the slope of the standard curve of the Cq values (coefficient of determination, R^2^ = 0.998). Testing of 10 replicates of 30 GU (9/10 positive) and 3 GU (5/10 positive) gave a cut-off of 30 GU (Minimum Information for Publication of Quantitative Real-Time PCR Experiments (MIQE) Guidelines) [24].

### *A. phagocytophilum* prevalence and geographical distribution

The overall prevalence of *A. phagocytophilum* in the collected ticks was 2.9 % (22/765) (Table 2). Of the ticks collected from dogs and cats 3.2 % (15/463) and 2.3 % (7/302), were positive for *A. phagocytophilum*, respectively (*p* = 0.46).

Among the adult female ticks, 2.8 % (19/690) were positive for *A. phagocytophilum*, while 4.3 % (3/69) of the adult male ticks were positive (*p* = 0.45). None of the four collected nymphs were positive for *A. phagocytophilum*.

Of the ticks collected in the three northernmost counties in Norway, 3.0 % (20/669) were positive for *A. phagocytophilum* (Nordland 2.8 % (18/647); Troms 9.1 % (2/22); the tick collected in Finnmark could not be included due to the pet’s recent stay outside the study area. Of the ticks collected in Telemark county, 2.1 % (2/96) were positive for *A. phagocytophilum* (Fig. 1). There was no significant difference in *A. phagocytophilum* prevalence in ticks collected in northern Norway compared to ticks collected in Telemark county (*p* = 0.63).

## Discussion

In this study we developed a real-time PCR assay targeting *gltA* with the purpose of direct detection of *A. phagocytophilum* in ticks. By using a large collection of ticks we give new insights into the epidemiology of *A. phagocytophilum* in ticks in northern Norway in relation to a southern Norwegian county (Telemark). We report here, for the first time, the presence of *A. phagocytophilum* in feeding ticks collected north of the Arctic Circle in Norway.

Previous studies have demonstrated the presence of *I. ricinus* in northern Norway as well as a high prevalence of *Borrelia burgdorferi* sensu lato in ticks in the region around the Arctic Circle [15, 20, 21]. Serological studies in two sheep flocks in Brønnøy found a high seroprevalence against *A. phagocytophilum*; 97 % and 100 %, respectively (Stuen et al*.*, unpublished data). In addition, a serological study in dogs from northern Norway found a seropositivity of 3 % (4/120); three of the dogs were from Brønnøy [25]. The presence of *A. phagocytophilum* has recently been reported in a smaller number of *I. ricinus* ticks collected by flagging vegetation in Brønnøy in the county of Nordland [15]. We found an overall prevalence of *A. phagocytophilum* in feeding ticks of 3.0 % in northern Norway, which is about the same prevalence as we found in Telemark county in southern Norway, and also comparable to the prevalence in *I. ricinus* in more southern parts of Europe [13]. The majority of the ticks in this study were collected in Nordland county (85 %), which includes the archipelago of Brønnøy, recognised for its high tick abundance [21]. Climate, day length, habitat, mixture of hosts, and host abundance seem to be favourable for *I. ricinus* in this area, and apparently also for maintenance of *A. phagocytophilum* in the tick population.

*Anaplasma phagocytophilum* is internationally regarded as an emerging tick-borne pathogen, and areas of endemicity include parts of North America, Europe and Asia [26]. These regions correspond to areas where the appropriate tick vectors are found (*I. ricinus* in Western Europe, *I. persulcatus* in Eastern Europe and Asia, *I. scapularis* and *I. pacificus* in Eastern and Western USA respectively) [27]. Tick population density and geographical distribution are affected by changes in climate, vegetation and host abundance [28], and continuous surveillance of ticks and tick-borne pathogens at their distribution limits are important for early detection of altered risk scenarios and threats to public health. Although increasingly detected, symptomatic HGA still appears to be rather rare in Europe [17, 29], as opposed to the situation in the USA where HGA is a notifiable disease with increasing incidence [30]. However, human seroprevalence in Europe varies between 2–28 % [29]. It is unclear whether the discrepancy between the seroprevalence and the low number of symptomatic cases results from underdiagnosis of HGA, asymptomatic serologic reactions, reduced virulence of circulating *A. phagocytophilum* strains in Europe or even infections that cause cross-reactive serologic responses [27]. In any case, *A. phagocytophilum* is endemic in Europe where it is the most widespread tick-borne pathogen in animals [31], and should therfore also be regarded as a potentially emerging human pathogen. Tick-exposed patients presenting with fever, leukopenia and elevated serum transaminases should have HGA included in the differential diagnoses [26].

The PCR method developed showed a high analytical sensitivity (30 GU) and efficiency (98.5 %). Based on the *in silico* analysis, the test is expected to be highly specific. It is rapid, and the results were found to be unambiguous and straightforward to interpret. We found this PCR assay to be useful for detection of *A. phagocytophilum* in ticks, and it may also be useful in clinical diagnostics in both human and veterinary medicine, but further evaluation of the method using clinical specimens will be needed.

*In silico* testing showed that 79/85 (93 %) of *Anaplasma phagocytophilum gltA* sequences are highly homologous to the primers and probe and will be efficiently detected. The same applies to a slightly more divergent sequence represented by isolate R33 (JX841254) [32] which, according to information presented in the sequence file, was detected in reindeer (*Rangifer tarandus*) imported to France. However, some far-eastern isolates are expected to be detected at low sensitivity, if at all. These include a group represented by strain KC478600, isolated from a rat (*Rattus norvegicus*) in southeastern China [33], and a single isolate, Khablx (AY339603), from *I. persulcatus* in the Russian far east [34].

## Conclusion

The present study implies that both humans and pets may contract anaplasmosis also in northern parts of Norway, and that physicians as well as veterinarians need to be aware of the disease. The PCR assay targeting *gltA* performed well, and may be useful in clinical diagnostics in the future, but may fail to detect certain far-eastern isolates.
